# Effect of Insulin Pump Use on Diabetic Ketoacidosis in Type 1 Diabetes Mellitus: A Matched Cohort Study

**DOI:** 10.3390/jcm10050898

**Published:** 2021-02-25

**Authors:** Abbas Alshami, Tiffany Purewal, Steven Douedi, Mohammed Alazzawi, Mohammad A. Hossain, Raquel Ong, Shuvendu Sen, Jennifer Cheng, Swapnil Patel

**Affiliations:** 1 Department of Medicine, Jersey Shore University Medical Center, Neptune, NJ 07753, USA; Abbas.Alshami@hmhn.org (A.A.); Tiffany.Purewal@hmhn.org (T.P.); Mohammed.Alazzawi@hmhn.org (M.A.); Mohammad.Hossain@hmhn.org (M.A.H.); Shuvendu.Sen@hmhn.org (S.S.); Swapnil.Patel@hmhn.org (S.P.); 2 Division of Endocrinology, Diabetes, and Metabolism, Jersey Shore University Medical Center, Neptune, NJ 07753, USA; Raquel.Ong@hmhn.org (R.O.); JenniferS.Cheng@hmhn.org (J.C.)

**Keywords:** diabetic ketoacidosis, diabetes mellitus, insulin, pump, injection

## Abstract

Background: Diabetic ketoacidosis (DKA) is a well-known complication of diabetes mellitus with a significantly high mortality if not immediately and properly treated. Therefore, strategies for prevention of DKA are ever so important when managing diabetes mellitus, especially in the non-compliant patient population. Previously studies have suggested insulin pump use to carry an increased risk of DKA compared to insulin injections, while European studies suggest the opposite. We aimed to perform a retrospective cohort study to determine the risk of DKA in insulin pump versus injection in the United States. Methods: We utilized the Healthcare Cost and Utilization Project National Inpatient Sample (HCUP-NIS) 2017 database, which represents a 20% sample of all payer hospitalizations in the United States. These hospitalizations were systematically selected by the Agency for Healthcare Resources and Quality (AHRQ) and we included all type 1 diabetes mellitus patients over the age of 18 who were on insulin, either pump or injections, in our study. Results: We found a total of 58,260 admissions for patients with type 1 DM. Of these, 7850 had insulin pump, 30,672 used insulin injection, and 19,738 had no prior insulin use. We found that insulin pump use, compared to injections, failed to predict a lower incidence of DKA in hospitalized patients. Conclusion: Although several studies from European countries have found a reduction of DKA risk with insulin pump use, in this study we found no clear significant difference in a United States-based study. While this may be possible due to different legislating and regulation organizations, further studies are warranted to further evaluate the benefit of either insulin dispensing modality.

## 1. Introduction

Diabetic ketoacidosis (DKA) is a life-threatening metabolic emergency in patients with diabetes mellitus (DM) [1]. When untreated, the mortality of DKA is higher than 90%; however, advancement in management and critical care services have decreased the mortality rate to less than 2% [2]. Nonetheless, DKA remains a significant cause of morbidity and mortality, with hospital admissions of 100,000 patients yearly in the United States [1]. Epidemiological factors such as age, sex, and socioeconomic status were found to be independent risk factors for DKA [3]. In addition, several precipitating factors for DKA were identified including infections, skipping insulin therapy, mode of insulin delivery, myocardial infarctions, stroke, depression, trauma, and substance abuse [3,4,5]. The mode of insulin delivery for patients leading to development of DKA is of particular interest and previous data have been controversial [6]. Prior to 1993, most studies suggested increased risk of DKA with insulin pump use [7]. Since insulin pump therapy uses rapid acting insulin only, if the pump or the pump site fails, there will be no insulin delivery, and blood glucose levels can rise rapidly, and ketones start to develop within 4–6 h. However, due to significant improvements of technology and more importantly, patient training, DKA has become a less prevalent complication with the use of insulin pump therapy [8]. Recently, large population-based observational studies in Europe on patients with type 1 diabetes mellitus (T1D) showed decreased risk of DKA with insulin pump use [9,10]. However, there is no population-based study, outside of controlled research settings, in the United States to investigate the effect of insulin pump use on DKA occurrence. Therefore, we sought to investigate the effect of insulin pump use on DKA incidence in hospitalized patients in the United States.

## 2. Materials and Methods

*Study Design/Settings*: This is a retrospective cohort study. We utilized the Healthcare Cost and Utilization Project National Inpatient Sample (HCUP-NIS) 2017 database, which represents a 20% sample of all payer hospitalizations in the United States. These hospitalizations were systematically selected by the Agency for Healthcare Resources and Quality (AHRQ), thus they represent all the hospitalizations in the United States in 2017. Notably, Encounters represent hospitalizations, not patients. The data collected in the database include demographic variables, admission diagnoses, procedures, type of insurance, geographical location, length of stay, and inpatient mortality, among others.

**Figure 1:**
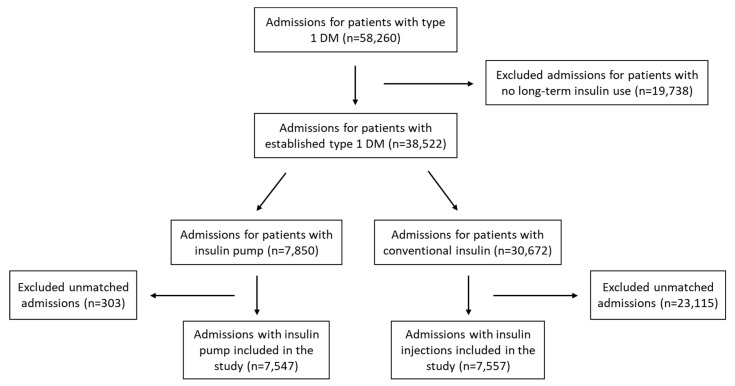
Flowchart of admissions selection in the study.

*Participants*: The selection process is summarized in Figure 1. All admissions for patients with type 1 DM were identified. To exclude patients whose initial presentation was diabetic ketoacidosis, we excluded patients who were not on insulin (injections or pump). To balance the study groups, exact matching was performed with 1:1 ratio, without replacement, matching for age, sex, race, and type of insurance (as a surrogate for socioeconomic status). 

*Variables*: Insulin pump represents any insulin pump machine irrespective of type. Similarly, T1D patients were included irrespective of severity or glycemic control. We aimed to primarily investigate the effect of insulin pump use on the incidence of DKA in hospitalized patients. Secondary outcomes include the effect of insulin pump use on in-hospital outcomes, such as mortality and length of stay in the hospital.

*Data measurement*: Age was reported in years. Six categories were reported for race, including white, black, Hispanic, Asian or pacific islander, native American, and other. Six categories were reported for type of primary insurance, including Medicare, Medicaid, private insurance, self-pay, no charge, and other. Medical conditions were identified through their international classification of diseases, 10th revision (ICD-10 codes) recorded in the discharge record for each hospitalization. For T1D, ICDs E10.2, E10.3, E10.4, E10.5, E10.6, E10.8, E10.9 were used. E10.1 was used for diabetic ketoacidosis. Assuming all patients with T1D should receive some sort of insulin therapy, we used long-term (current) use of insulin ICD, Z794, as a surrogate for established T1D, as opposed to patients with no previous diagnosis initially presenting to the hospital with DKA. We identified patients with insulin pump using ICD for presence of insulin pump (external) (internal), Z9641, or ICDs for insulin pump complications, T856 or T8572.

*Ethical Considerations*: The institutional review board (IRB) is not required for studies utilizing the national inpatient samples, as they fall under the “limited data sets” category exempted by the HIPAA privacy regulations (http://privacyruleandresearch.nih.gov (accessed on 17 January 2021)). The study was conducted in agreement with the principles of the Declaration of Helsinki.

*Statistical Methods*: Non-parametric continuous and categorical variables were described as median with interquartile range and frequencies, as appropriate. Chi-square was used to compare categorical variables, and independent sample *t*-test was used to compare continuous variables. Missing data were examined for four variables (age, sex, race, type of insurance), and they were missing 1, 2, 2251, and 127 values, respectively. Assuming missing data were missing at random (MAR), multiple imputation model using the fully conditional specification (FCS) method was utilized to create 5 imputations with 10 iterations, which seemed to provide sufficient convergence, to impute missing values for the four aforementioned variables. In addition to these four variables, other auxiliary variables (emergency service indicator, census division of the hospital, NIS hospital number, patient location, and median household income for patient’s zip code) were used as predictors to increase the precision of the imputation process. Then, within each of the 5 imputed data sets, the Exact Matching (EM) method was performed to achieve 1:1 matching, for age, sex, race, and type of insurance, without replacement, and groups were compared after matching for any imbalances. Since the database is large and the matching was done for few variables and to achieve more precise estimation, matched participants in each imputed set were merged into a new stratum [11]. Then, the five strata were pooled, and standard logistic regression model (enter method) with adjustment for matched variables was utilized. Correlation matrix was created and examined for any potential multicollinearity. All analyses were done using IBM SPSS Statistics^TM^ version 25.0 (IBM Corporation, Artmonk, NY, USA). An alpha value (*p*) of 0.05 was used to ascertain statistical significance.

## 3. Results

We found a total of 58,260 admissions for patients with type 1 DM. Of these, 7850 had insulin pump, 30,672 used insulin injection, and 19,738 had no prior insulin use. The number of matched cases slightly differed between the imputation sets, with pooled stratum composed of 7557 admissions with conventional insulin and 7547 admissions with insulin pump (Figure 1).

*Baseline Characteristics*: Baseline characteristics significantly differed between patients using insulin pump and patients on conventional insulin therapy (Table 1). After matching, there was no statistically significant differences in age, sex, race, or type of insurance between study groups (Table 2). 

*In-hospital incidence of DKA*: In a bivariate analysis of the pre-matching sample, 24.5% of admissions for patients with T1D on conventional insulin therapy were for DKA (n = 7501), as opposed to 20.8% of admission for patients using insulin pump (n = 1629) (*p* < 0.001). However, in the post-matching sample, 20.9% and 20.4% of admissions were for DKA in insulin pump and conventional insulin therapy groups, respectively, and the difference was not statistically significant, *p*-values were insignificant across all imputation sets and ranged from 0.268 to 0.534. In a multivariate analysis of the pooled matched data (Table 3), insulin pump use also failed to predict a lower incidence of DKA in hospitalized patients.

## 4. Discussion

The first commercial insulin pump was introduced in 1983 in an attempt to further optimize blood glucose levels after growing evidence had supported that achievement of near-normal blood glucose levels result in reduction of diabetes-related complications [12]. However, insulin pumps, as any technical devices, are subjective to malfunctions which can result in lack of insulin delivery and occurrence of DKA. Indeed, a meta-analysis conducted by Weissberg-Benchell et al. demonstrated that studies prior to 1993 showed an increased incidence of DKA in patients using insulin pumps [7]. However, advancements of technologies implemented in newer insulin pumps have led to more reliable and precise machines, such as continuous glucose monitor and closed-loop systems. Another significant component in insulin pump failures is human factors and or user errors, which can result in many adverse effects, yet can be improved with proper training and the learning curve related to the use of insulin pump. One study showed that patients seem to do better with an insulin pump after one year of use, with improved glycemic control, and decreased incidence of hypoglycemia [8].

In this study, we found that the use of continuous insulin infusion did not result in lower incidence of DKA over the use of conventional insulin injections in contrast to studies in other countries that have shown that the use of an insulin pump not only reduces the risk of DKA, but also plays a significant role in improved glycemic control, with lower hemoglobin A1c levels, and decreased incidence of hypoglycemia [8,9,10]. Legal regulatory authorities could be the reason behind such difference [13,14]. There appears to be fundamental differences between the regulatory authorities in the United States (US) and Europe [14]. In the US, insulin pumps are classified as Class II (moderate risk) devices by the Food and Drug Administration (FDA), while insulin pumps with continuous glucose monitoring are classified as Class III (higher risk), which translates to a thorough and lengthy approval process to ensure safety of these devices. In Europe, continuous glucose monitoring devices are classified as Class IIa (moderate risk) and pumps are classified as Class IIb (moderate risk), which differs from the higher risk classification in the US. Class IIa/b devices must undergo a “conformity assessment procedure”, where a Conformite Europeene (CE) mark must be obtained for medical device utilization within European clinical practice. This is accomplished through a “notified body”. These notified bodies are independent commercial organizations who obtain some financial support through fees paid by device manufacturing companies [15]. When a company plans to market a new device, they have the ability to choose any “notifying body”, who then decides if the device meets required specifications. Once the notifying body issues the certificate, the company can then market the device throughout Europe. Requirements for standardized trials and studies in Europe are not enforced and health authorities do not have a post-marketing surveillance system [15]. Furthermore, in the US, the FDA has a structured process in regard to reporting adverse events associated with medical devices and they have the authority to enforce post-marketing surveillance of medical devices as they see fit [14]. These adverse events are then made public via the Manufacturer and User Facility Device Experience (MAUDE) database. A comparable database does not exist in Europe and data regarding adverse events are not publicly available. The overall regulations in Europe possibly provide a lower level of surveillance when approving devices for clinical practice as opposed to the regulations that exist in the US [13,14,15]. Therefore, insulin pumps and continuous glucose monitors are perhaps more utilized in clinical practice in Europe than in the US. This may explain why our study did not find a decrease incidence in DKA when using continuous insulin infusion compared to conventional injections in the US. It is possible that medical devices are more freely utilized into clinical practice in Europe, and therefore the efficacy of insulin pumps is more apparent. Another possible explanation is that adverse events relating to insulin pumps are more readily accessible in the US; owing to the difference is findings between countries [14]. Further studies may be warranted in order to ascertain the explanation behind these disparities.

Insulin pump therapy offers the advantage of flexibility and improved quality of life [12]. Perhaps, over time, the improvement in technology of the insulin pump has reduced the complications. There may also be a learning curve related to the use of an insulin pump. In another prior study, patients seem to do better with an insulin pump after one year of use, with improved glycemic control, and decreased incidence of DKA and hypoglycemia [8]. However, insulin pump therapy is more costly compared to multiple daily injections [14]. Continuous insulin infusion therapy costs $3923 more annually than those on daily injections from increased expense due to office visits, medications, and inpatient costs [14]. Further studies should also consider other possible complications with insulin pump therapy, including malfunction, localized infections and challenges related to prolonged fasting and nothing by mouth (NPO) status [12]. Our study did not look into which factors would show improved outcomes in patients on insulin pump therapy. Further investigation should be done with specific comorbidities to see which patients’ insulin pump therapy would suit the best.

Large sample size represents a strength in our study. However, we recognize several limitations, including, but not limited to, the utilized data is administrative and primarily gathered for billing purposes but did not include causes or reasons for developing DKA in the patients reported or pump brand or type. Several variables were determined by their relevant ICD-10 codes, which can introduce selection bias, depending on the accuracy of the codes’ abstractors. In addition, we could not define ketosis prone type 2 diabetes, identify the duration of T1D, or the level of glycemic control which could also affect the results. Further studies that include these factors are warranted.

## 5. Conclusions

Although several studies from European countries have found significant benefit in DKA management as well as improved glycemic control compared to conventional injections, in this study we found no clear significant difference in regard to DKA incidence in the United States. While this is likely due to different legislations and regulations, compliance and ease of use are factors favoring insulin pump use over injections, although patients’ expenses also play a role in willingness to use an insulin pump. Despite a large sample size and no clinical significance in regard to DKA incidence, several further studies are warranted to examine the benefit of insulin pumps versus injections in the United States.

## Figures and Tables

**Table 1:** Comparison of Baseline Characteristics of Insulin Pump Group and Conventional Insulin Group before Matching.

	Insulin Pump Group	Conventional Insulin Group	*p*-Value
	Median (IQR) or N (%)	Median (IQR) or N (%)	
Age (years)	42 (14–70)	36 (15–57)	
Sex			<0.001
Male	2925 (37.3)	14,625 (47.7)	
Female	4925 (62.7)	16,047 (52.3)	
Race			<0.001
White	6559 (83.6)	19,080 (62.2)	
Black	670 (8.5)	7002 (22.8)	
Hispanic	399 (5.1)	3230 (10.5)	
Asian or Pacific Islander	58 (0.7)	423 (1.4)	
Native American	33 (0.4)	277 (0.9)	
Other	131 (1.7)	660 (2.2)	
Type of Insurance			<0.001
Medicare	2760 (35.2)	11,478 (37.4)	
Medicaid	1253 (16)	9750 (31.8)	
Private Insurance	3575 (45.5)	6862 (22.4)	
Self-pay	108 (1.4)	1718 (5.6)	
No charge	8 (0.1)	142 (0.5)	
Other	146 (1.9)	722 (2.4)	
Total	7850	30,672	

Abbreviations: DM1, diabetes mellitus type 1; DKA, diabetic ketoacidosis; IQR, interquartile range.

**Table 2:** Comparison of Baseline Characteristics of Insulin Pump Group and Conventional Insulin Group After Matching.

	Matched Cohort		*p* Value *	Unmatched Cohort		*p* Value *
	Median (IQR) or *N* (%)			Median (IQR) or *N* (%)		
	IP Group	CI Group		IP Group	CI Group	
Age (years)	46 (31–60)	46 (31–60)	0.894			
Sex			0.959			<0.001
Male	2857 (37.9)	2862 (37.9)		66 (22.1)	11,762 (50.9)	
Female	4690 (62.1)	4695 (62.1)		232 (77.9)	11,353 (49.1)	
Race						<0.001
White	6379 (84.5)	6382 (84.4)	1	176 (59.1)	12,708 (55)	
Black	658 (8.7)	662 (8.8)		12 (4)	6324 (27.4)	
Hispanic	371 (4.9)	374 (5)		26 (8.7)	2853 (12.3)	
Asian or Pacific Islander	35 (0.5)	35 (0.4)		23 (7.7)	389 (1.7)	
Native American	17 (0.2)	17 (0.2)		16 (5.4)	264 (1.2)	
Other	87 (1.2)	87 (1.2)		45 (15.1)	577 (2.4)	
Type of Insurance						<0.001
Medicare	2741 (36.3)	2742 (36.3)	0.999	17 (5.7)	8735 (37.8)	
Medicaid	1231 (16.3)	1235 (16.3)		22 (7.4)	8512 (36.8)	
Private Insurance	3366 (44.6)	3370 (44.6)		207 (69.4)	3493 (15.1)	
Self-pay	97 (1.3)	98 (1.3)		10 (3.4)	1623 (7)	
No charge	4 (0.1)	4 (0.1)		4 (1.3)	138 (0.6)	
Other	109 (1.4)	109 (1.4)		38 (12.8)	614 (2.7)	
Total	7547	7557		298	23,115	

Abbreviations: IP, insulin pump; CI, conventional insulin. * The lowest *p*-value among the five imputations are reported.

**Table 3:** Multivariate logistic regression model to predict occurrence of diabetic ketoacidosis.

	OR	95% CI for OR	*p*-Value
Age	0.985	0.982–0.987	<0.001
Female Sex	1.160	1.065–1.263	0.001
Insurance–Medicare	1		Ref
Insurance–Medicaid	1.236	1.085–1.408	0.001
Insurance–Private insurance	0.706	0.635–0.785	<0.001
Insurance–Self-pay	2.009	1.476–2.735	<0.001
Insurance–No charge	0.543	0.065–4.526	0.57
Insurance–Other	0.78	0.541–1.124	0.18
Race–white	1		Ref
Race–black	1.172	1.018–1.348	0.03
Race–Hispanic	0.853	0.707–1.029	0.10
Race–Asian or Pacific Islander	0.987	0.504–1.933	0.97
Race–Native American	0.758	0.308–1.868	0.55
Race–Other	0.724	0.478–1.097	0.13
Insulin pump	1.038	0.957–1.125	0.37

## Data Availability

The authors declare that data supporting the findings of thisstudy are available within the article.
